# Supplementary material for: Caenorhabditis elegans to Model the Capacity of Ascorbic Acid to Reduce Acute Nitrite Toxicity under Different Feed Conditions: Multivariate Analytics on Behavioral Imaging
Source: Int J Environ Res Public Health. 2021 Feb 20;18(4):2068. doi: 10.3390/ijerph18042068 (PMC7923789; doi:10.3390/ijerph18042068)
Supplement: Supplementary file 1 [file ijerph-18-02068-s001.pdf]

Supplementary material

1. Video preprocessing procedure

Firstly, videos are converted into image sequences. Next a background estimation procedure was run to obtain an image representing the background; i.e. an image with no nematode (Figure 1c). This background image was obtained as follows:

$$background(x,y) = median(\{imagei(x,y) : i=1\ldots N\}) \qquad (1)$$

where imagei (x,y) represents the (x,y) pixel of the i-th image extracted from the video and N the total number of images. The result was an image with the median values of each pixel, called the median image (Gonzalez et al., 2013). The median image filtered the temporal pixel variations by offering, in this case, an image with no nematodes. The background image was then used to estimate the regions of interest (ROIs) in the images; i.e. the Petri plate areas where the nematode moved. First a sobel preprocess (Gonzalez et al., 2013) was applied to compute borders. Then the image was binarised and the circular binary blobs representing the Petri plates were located (Figure 1d). Having defined the ROIs, a mask image was obtained to limit the areas where specimens were searched. Finally, a threshold (Ψ) was computed from the mean pixel value in the ROIs to automate specimen location among the different lighting conditions. Having obtained the background image, the mask image and threshold Ψ were computed, and the extracted images were processed as shown in Figure 1g and k. For each image, the background was removed by means of subtraction and the mask was applied to remove information from the ROIs. The processed images were then binarised using the computed threshold Ψ and the binary blobs were analyzed and filtered by area, leaving only the located specimens. Each ROI was then cropped from the image and processed to obtain the required features.

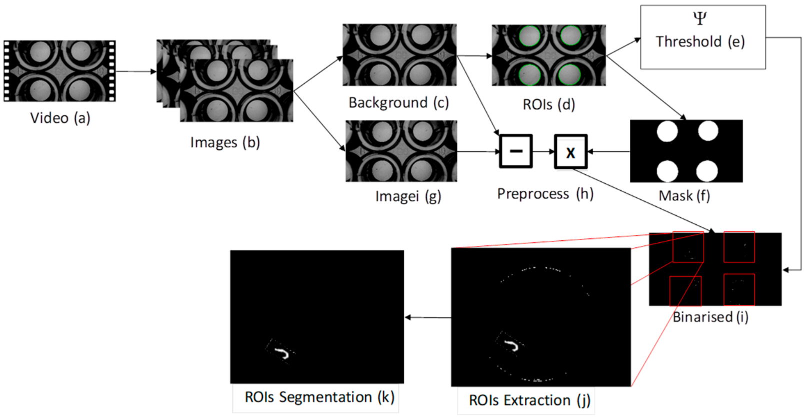

Figure S1. Video processing. Nematode segmentation.

2. Behavioural analysis

Histograms were computed as follows, where L={li : i=1…N} is the set of worm length values and N is the number of captured frames and, thus, the number of descriptor values. An lm value was computed as:

$$Lm = max(\{li: i = 1 \ldots N\}) \tag{2}$$

and a normalised version of L, Ln, was obtained,

$$Ln = \left\{100 \cdot \left(\frac{li}{lm}\right): i = 1 \ldots N\right\} \tag{3}$$

Finally, the histogram of descriptor L, HL, was calculated as Hl={hj : j=0…99} where,

$$hj = \frac{|\{l \epsilon Ln: j < l \leq j+1\}|}{N} \tag{4}$$
